# An updated clinical prediction model of protein-energy wasting for hemodialysis patients

**DOI:** 10.3389/fnut.2022.933745

**Published:** 2022-12-06

**Authors:** Si Chen, Xiaoyan Ma, Xun Zhou, Yi Wang, WeiWei Liang, Liang Zheng, Xiujuan Zang, Xiaobin Mei, Yinghui Qi, Yan Jiang, Shanbao Zhang, Jinqing Li, Hui Chen, Yingfeng Shi, Yan Hu, Min Tao, Shougang Zhuang, Na Liu

**Affiliations:** ^1^Department of Nephrology, Shanghai East Hospital, Tongji University School of Medicine, Shanghai, China; ^2^Key Laboratory of Arrhythmias of the Ministry of Education of China, Research Center for Translational Medicine, Shanghai East Hospital, Tongji University School of Medicine, Shanghai, China; ^3^Department of Nephrology, Shanghai Songjiang District Central Hospital, Shanghai, China; ^4^Department of Nephrology, Shanghai Gongli Hospital, Shanghai, China; ^5^Department of Nephrology, Shanghai Punan Hospital, Shanghai, China; ^6^Department of Medicine, Rhode Island Hospital and Alpert Medical School, Brown University, Providence, RI, United States

**Keywords:** protein-energy wasting, hemodialysis, prediction model, nomogram, impact factors

## Abstract

**Background and aim:**

Protein-energy wasting (PEW) is critically associated with the reduced quality of life and poor prognosis of hemodialysis patients. However, the diagnosis criteria of PEW are complex, characterized by difficulty in estimating dietary intake and assessing muscle mass loss objectively. We performed a cross-sectional study in hemodialysis patients to propose a novel PEW prediction model.

**Materials and methods:**

A total of 380 patients who underwent maintenance hemodialysis were enrolled in this cross-sectional study. The data were analyzed with univariate and multivariable logistic regression to identify influencing factors of PEW. The PEW prediction model was presented as a nomogram by using the results of logistic regression. Furthermore, receiver operating characteristic (ROC) and decision curve analysis (DCA) were used to test the prediction and discrimination ability of the novel model.

**Results:**

Binary logistic regression was used to identify four independent influencing factors, namely, sex (*P* = 0.03), triglycerides (*P* = 0.009), vitamin D (*P* = 0.029), and NT-proBNP (*P* = 0.029). The nomogram was applied to display the value of each influencing factor contributed to PEW. Then, we built a novel prediction model of PEW (model 3) by combining these four independent variables with part of the International Society of Renal Nutrition and Metabolism (ISRNM) diagnostic criteria including albumin, total cholesterol, and BMI, while the ISRNM diagnostic criteria served as model 1 and model 2. ROC analysis of model 3 showed that the area under the curve was 0.851 (95%CI: 0.799–0.904), and there was no significant difference between model 3 and model 1 or model 2 (all *P* > 0.05). DCA revealed that the novel prediction model resulted in clinical net benefit as well as the other two models.

**Conclusion:**

In this research, we proposed a novel PEW prediction model, which could effectively identify PEW in hemodialysis patients and was more convenient and objective than traditional diagnostic criteria.

## Introduction

Chronic kidney disease (CKD) is a global public health problem. CKD progresses to a terminal stage termed as end-stage renal disease (ESRD), requiring renal replacement therapy (hemodialysis, peritoneal dialysis, and kidney transplantation). According to the Renal Data System, the number of patients with ESRD requiring renal replacement therapy is increasing year by year in the United States, reaching nearly 7,50,000 in 2019 (1). Sixty-three percent of these individuals receive maintenance hemodialysis (MHD) (1). Despite improving survival rates, only 57% of MHD patients are still alive 3 years after their first treatment (1). The main reasons for such a high-mortality rate are cardiovascular diseases (2). However, these factors do not fully explain the increased risk of mortality. With the progression of CKD, patients are accompanied by nutritional disorder and muscle catabolism, leading to protein-energy wasting (PEW) (3–5). PEW is a multifactorial, maladaptive metabolic state characterized by a loss of body protein mass and energy reserves and is a major cause of high morbidity and mortality in patients with CKD (6, 7).

It has been revealed that PEW is prevalent in patients with CKD. Based on a meta-analysis, the global prevalence of PEW is estimated to be 11–54% among patients with CKD (stages 3–5) and 28–54% among dialysis patients (8). Up to now, the mechanism of PEW is still unclear, and the current physiopathological mechanisms of PEW mainly include inflammation, insulin resistance, oxidative stress, hormone dysregulation, and metabolic acidosis (9–11). Low-grade inflammation is a common hallmark of CKD (12). Recent studies have demonstrated that inflammatory cytokines such as interleukin-18 (IL-18), interleukin-6 (IL-6), and galectin-3 are critically involved in PEW (13, 14). Persistent inflammation may damage the structure and function of different tissues, thus destroying the normal inter-organ crosstalk and leading to metabolic disorders (14). Furthermore, inflammation can lead to adipose tissue browning, muscle atrophy, and increased resting energy expenditure (REE), which ultimately lead to PEW (15). Recent basic and clinical studies demonstrate that chronic kidney disease–mineral and bone disorder (CKD–MBD) directly induces inflammation and PEW, and high circulating levels of parathyroid hormone (PTH) have been proven to induce the inflammatory response that leads to PEW (16). PTH can also increase adipose tissue browning and REE, which explains the clinical association between secondary hyperparathyroidism and PEW in hemodialysis patients (17–20). In addition, N-terminal pro-B-type natriuretic peptide (NT-proBNP), as a robust biomarker of muscle wasting, is elevated in MHD patients and can independently predict PEW (21–23).

The International Society of Renal Nutrition and Metabolism (ISRNM) recommends four main categories to be recognized for the diagnosis of PEW (24): low levels of biochemical criteria (i.e., albumin, prealbumin, and cholesterol); low body weight, reduced total body fat, or weight loss; a decrease in muscle mass (i.e., muscle wasting and reduced mid-arm muscle circumference area); and low protein or energy intake. At least three out of the four listed categories must be satisfied to diagnose kidney disease-related PEW. However, the identification and diagnosis of PEW rely on the clinical judgment process, depending on meeting multiple criteria, including serum biochemistry, body weight status, muscle mass prediction, dietary energy, and protein intake (10). In particular, estimating dietary intake for patients with chronic diseases is difficult, and the data obtained may not be reliable (25, 26). In this research, we conducted the analysis of independent influencing factors of PEW and proposed a novel PEW prediction model, in order to make the diagnosis of PEW more objective and convenient, thus improving the life quality and reducing the mortality of MHD patients.

## Materials and methods

### Study design and participants

This was a cross-sectional study in MHD patients from four different medical centers in Shanghai, including Shanghai East Hospital, Shanghai Songjiang District Central Hospital, Shanghai Punan Hospital, and Shanghai Gongli Hospital. The study recruited MHD patients according to the following inclusion criteria: age range 18–75 years; maintenance hemodialysis for over 6 months; and consented to participate in all aspects of the study. Patients with the following conditions were excluded: pregnancy; thyroid dysfunction; corticosteroid or immunosuppressive medication; systemic infections, cardiovascular events, operations, trauma, and tumors for which a patient had received radiotherapy or chemotherapy within 3 months; active communicable diseases; patients enrolled in other clinical studies; poor compliance; and patients who underwent nutritional interventions. A total of 380 participants were ultimately included in this study, including 190 participants from Shanghai East Hospital, 92 participants from Shanghai Songjiang District Central Hospital, 60 participants from Shanghai Punan Hospital, and 38 participants from Shanghai Gongli Hospital. The Ethics Committee of Shanghai East Hospital approved the study protocol and adhered to the Declaration of Helsinki. Each patient provided written informed consent to participate in the study. This trial was registered at ClinicalTrials.gov (ChiCTR2000038127).

### Assessment of protein-energy depletion

According to the diagnostic criteria proposed by the ISRNM in 2008 (24), at least three out of the four listed categories must be satisfied for the diagnosis of kidney disease-related PEW (each criterion should be documented on at least three occasions, preferably 2–4 weeks apart): (1) serum chemistry: serum albumin <38 g/L, serum prealbumin <0.3 g/L, or serum cholesterol <1 g/L; (2) body mass: body mass index (BMI) <23 kg/m^2^, unintentional 5% weight loss over 3 months or 10% weight loss over 6 months, and total body fat percentage <10%; (3) muscle mass: reduced 5% muscle mass over 3 months or 10% over 6 months, reduction of mid-arm muscle circumference (MAMC) area over 10% in relation to 50th percentile of reference population, and creatinine appearance; and (4) dietary protein intake (DPI): unintentional low DPI < 0.80 g/kg per day for at least 2 months for dialysis patients or DPI < 0.60 g/kg per day for patients with CKD stages 2–5.

### Demographic and laboratory measurements

Demographic and clinical data including age, sex, education level, height, weight, primary renal disease, comorbidities (hypertension, diabetes, hyperlipidemia, stroke, and cardiovascular disease), systolic blood pressure, and diastolic blood pressure were collected. BMI was calculated by dividing the dry weight of dialysis patients by their height^2. MAMC was calculated by using the following formula: MAMC = arm circumference (mm)–3.14 * triceps skin-fold thickness (mm) (27). We used a 3-day dietary questionnaire to record the dietary intake of each patient for three consecutive days (including two working days and one weekend) to estimate their DPI (3).

Blood samples were collected following an overnight fast (before dialysis). Biochemistry data including serum albumin (g/L), serum prealbumin (mg/L), serum bilirubin (μmol/L), alanine aminotransferase (U/L), aspartate aminotransferase (U/L), serum creatinine (μmol/L), blood urea nitrogen (mmol/L), serum uric acid (μmol/L), triglyceride (TC, mmol/L), total cholesterol (TG, mmol/L), high-density lipoprotein cholesterol (HDL-c, mmol/L), low-density lipoprotein cholesterol (LDL-c, mmol/L), fasting blood glucose (mmol/L), serum calcium (mmol/L), serum magnesium (mmol/L), serum phosphorous (mmol/L), serum iron (μmol/L), ferritin (ng/mL), PTH (pg/mL), vitamin D (ng/mL), NT-proBNP (ng/L), lymphocyte count (10^9/L), hemoglobin (g/L), C-reactive protein (CRP, mg/L), and urea clearance index (Kt/V urea) were collected. Serum albumin, bilirubin, alanine aminotransferase, aspartate aminotransferase, creatinine, blood urea nitrogen, uric acid, TC, TG, HDL-c, LDL-c, fasting blood glucose, serum calcium, serum magnesium, serum phosphorous, serum iron, ferritin, hemoglobin, CRP, and PTH were measured by enzymatic colorimetry; prealbumin was measured by immunoturbidimetry; vitamin D was measured by competition method; and NT-proBNP was measured by double antibody sandwich method. All central laboratory data detection methods were unified.

### Statistical analysis

Continuous variables were presented as mean and standard deviation or median and interquartile ranges. Categorical variables were presented as counts and percentages. The independent sample *t*-test, Mann–Whitney *U* test, or χ^2^ test was used to compare the differences between PEW and non-PEW participants. The data were Ln-transformed to reduce skew. Then, univariate logistic regression was used to identify potential factors; factors with an associated *P*-value of less than 0.05 were entered into both forward and backward conditional multivariable logistic regression procedures. The prediction strength was quantified as odds ratios (ORs) with 95% confidence intervals (CIs). The function of the “rms” package in R software was invoked to establish the nomogram model based on the results of multivariable logistic regression.

Model stability was assessed *via* bootstrap analysis (plots of predicted versus observed outcomes, 1,000 bootstrap samples), a graphic representation of the relationship between the observed outcome frequencies and the predicted probabilities. The predictions should fall on a 45-degree diagonal line in a well-calibrated model. Model discrimination was assessed by the receiver operating characteristic (ROC) and quantified by the area under the ROC curve. DCA was used to estimate and compare clinical benefits between different prediction models. Introducing “threshold probability” to trigger medical intervention under the same threshold probability, if the nomogram brings a higher net benefit to patients, its clinical practicability will be better. All probabilities were two-tailed, and the level of significance was set at 0.05. Statistical analysis was performed using SPSS (version 23.0) and RStudio (version 2021.09.1 + 372).

## Results

### Basic characteristics

**FIGURE 1:**
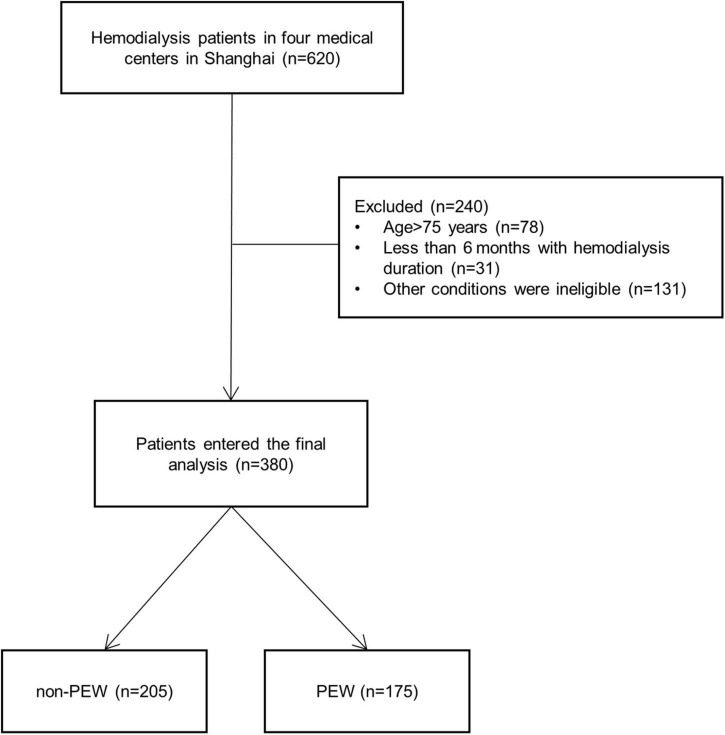
Flowchart of the protein-energy wasting (PEW) study. Six hundred and twenty hemodialysis patients from four centers in Shanghai were included in the study, and 240 patients were excluded according to the criteria. Three hundred and eighty patients were finally enrolled, consisting of 175 PEW patients and 205 non-PEW patients. PEW, protein-energy wasting.

Six hundred and twenty hemodialysis patients from four centers in Shanghai were included in the study, and 240 patients were excluded according to the inclusion criteria (Figure 1). Three hundred and eighty patients were finally enrolled and were partitioned into two groups by the diagnostic criteria of PEW (24): PEW group (*n* = 175, 98 male and 77 female patients) and non-PEW group (*n* = 205, 141 male and 64 female patients). The criteria met by the 175 patients diagnosed with PEW are listed in Table 1. The prevalence of PEW was 46.05%. The female patients were presented with a significantly increased incidence of PEW compared with the male patients (*P* < 0.05). The etiologies of participants are given in Table 2: hypertensive nephropathy (10.79%), diabetic nephropathy (24.26%), chronic glomerulonephritis (31.58%), nephrotic syndrome (2.89%), IgA nephropathy (2.10%), purpura nephritis (0.79%), obstructive nephropathy (0.79%), polycystic renal disease (3.95%), and unknown etiology (21.84%). There was no difference between the etiologies of the PEW group and the non-PEW group (*P* = 0.676).

**TABLE 1:** The criteria met by the 175 patients diagnosed with protein-energy depletion (PEW).

➀	➁	➂	➃	PEW (n)
Yes	Yes	Yes	No	83
Yes	Yes	No	Yes	58
Yes	No	Yes	Yes	64
No	Yes	Yes	Yes	81
Yes	Yes	Yes	Yes	37

➀: serum chemistry: serum albumin < 38 g/L, serum prealbumin < 0.3 g/L, or serum cholesterol < 1 g/L; ➁: body mass: body mass index (BMI) < 23 kg/m^2^, unintentional 5% weight loss over 3 months, or 10% weight loss over 6 months, total body fat percentage < 10%; ➂: muscle mass: reduced 5% muscle mass over 3 months or 10% over 6 months, reduction of mid-arm muscle circumference (MAMC) area over 10% in relation to 50th percentile of the reference population, creatinine appearance; ➃: dietary protein intake (DPI): unintentional low DPI < 0.80 g/kg per day for at least 2 months for dialysis patients or DPI < 0.60 g/kg per day for patients with CKD stages 2–5.

**TABLE 2:** Primary diseases of the patients.

Primary diseases, *n* (%)	Total (*n* = 380)	PEW (*n* = 175)	Non-PEW (*n* = 205)	χ^2^	*p*
				5.742	0.676
Hypertensive nephropathy	41 (10.79)	21 (12)	20 (9.76)		
Diabetic nephropathy	96 (24.26)	43 (24.57)	53 (25.85)		
Chronic glomerulonephritis	120 (31.58)	53 (30.29)	67 (32.68)		
Nephrotic syndrome	11 (2.89)	7 (4)	4 (1.95)		
IgA nephropathy	8 (2.10)	4 (2.29)	4 (1.95)		
Purpura nephritis	3 (0.79)	2 (1.14)	1 (0.49)		
Obstructive nephropathy	3 (0.79)	1 (0.57)	2 (0.98)		
Polycystic renal disease	15 (3.95)	7 (4)	8 (3.90)		
Etiology unknown	83 (21.84)	37 (21.14)	46 (22.44)		

PEW, protein-energy wasting.

The clinical characteristics and biochemical data of all participants are summarized in Table 3. The median age of patients was 63 (interquartile range 55–68) years in the PEW group and 61 (interquartile range 52–68) years in the non-PEW group. There were significant differences between the two groups regarding sex, education level, monthly frequency of HD, diabetes, and hyperlipidemia (all *P* < 0.05). In terms of the biochemical parameters, albumin, prealbumin, TG, creatinine, blood urea nitrogen, blood uric acid, TC, HDL-c, LDL-c, serum phosphorous, serum iron, vitamin D, NT-proBNP, hemoglobin, and Kt/V urea were significantly different between the PEW group and the non-PEW group (all *P* < 0.05).

**TABLE 3:** General information and clinical characteristics of participants.

	Total (*n* = 380)	PEW (*n* = 175)	Non-PEW (*n* = 205)	t/z/χ^2^	*P*
Age (years)	63 (53∼68)	63 (55∼68)	61 (52∼68)	–0.87	0.385
Male sex, *n*(%)	239 (62.89)	98 (56.00)	141 (68.78)	6.61	0.010
Monthly frequency of HD	8.90 ± 2.175	9.20 ± 1.91	8.64 ± 2.35	–1.99	0.047
Education level, *n*(%)	−	−	-	13.80	0.008
Primary school	176 (46.32)	95 (54.29)	81 (39.51)		
Junior school	102 (26.84)	44 (25.14)	58 (28.29)		
High school	72 (18.95)	30 (17.14)	42 (20.49)		
College	22 (5.79)	5 (2.86)	17 (8.29)		
Above college	8 (2.11)	1 (0.57)	7 (3.41)		
Hypertension, *n*(%)	311 (91.20)	147 (90.18)	164 (92.13)	0.40	0.525
Diabetes, *n*(%)	178 (50.71)	99 (60.00)	79 (42.47)	10.75	0.001
Hyperlipidemia, *n*(%)	251 (66.40)	92 (52.87)	159 (77.94)	26.45	0.000
Stroke, *n*(%)	22 (6.47)	14 (8.70)	8 (4.47)	2.50	0.114
CVD, *n*(%)	59 (17.35)	32 (19.88)	27 (15.08)	1.36	0.244
SBP (mmHg)	143.05 ± 24.07	143.74 ± 25.98	142.51 ± 22.54	–0.44	0.660
DBP (mmHg)	79.37 ± 12.69	79.73 ± 11.91	79.09 ± 13.29	–0.17	0.866
Dialysis duration time (months)	59.00 (29.00∼118.50)	60.00 (29.50∼126.50)	58.50 (29.00∼117.50)	–0.29	0.775
BMI (kg/m^2^)	22.65 ± 3.60	20.81 ± 2.72	24.23 ± 3.51	–9.91	0.000
MAMC (cm)	21.58 ± 3.26	19.76 ± 2.41	23.13 ± 3.07	11.99	0.000
DPI (g/kg/day)	0.66 ± 0.14	0.60 ± 0.11	0.71 ± 0.14	–8.01	0.000
Albumin (g/L)	39.80 ± 4.06	38.40 ± 3.84	41.10 ± 3.86	–6.72	0.000
Pre-albumin (mg/L)	313.76 ± 92.5	284.50 ± 86.98	344.06 ± 88.46	–6.15	0.000
Total bilirubin (μmol/L)	6.16 ± 2.72	6.39 ± 3.21	5.97 ± 2.21	–1.08	0.280
ALT (U/L)	10.67 ± 8.66	11.04 ± 10.23	10.35 ± 7.04	–0.18	0.861
AST (U/L)	12.18 ± 9.14	12.91 ± 11.25	11.56 ± 6.80	–0.98	0.327
BUN (mmol/L)	25.12 ± 6.15	23.82 ± 5.54	26.22 ± 6.44	–4.07	0.000
Scr (μmol/L)	989.40 (815.00∼1148.00)	917.18 (733.00∼1078.00)	1069.00 (897.09∼1215.28)	–5.33	0.000
SUA (μmol/L)	449.49 (393.75∼511.87)	440.48 (384.00∼494.44)	466.00 (403.00∼520.27)	–2.62	0.009
TG (mmol/L)	1.59 (1.04∼2.51)	1.39 (0.90∼2.11)	1.90 (1.26∼2.74)	–4.84	0.000
TC (mmol/L)	3.40 (2.92∼4.27)	3.49 (2.79∼4.10)	3.72 (3.05∼4.45)	–2.92	0.004
HDL-c (mmol/L)	0.92 (0.75∼1.11)	1.02 (0.85∼1.16)	0.87 (0.71∼1.04)	–4.31	0.000
LDL-c (mmol/L)	1.89 (1.46∼2.46)	1.83 (1.30∼2.31)	1.92 (1.52∼2.71)	–2.31	0.021
Calcium (mmol/L)	2.31 (2.18∼2.48)	2.33 (2.18∼2.48)	2.31 (2.17∼2.48)	–0.18	0.854
Magnesium (mmol/L)	1.09 (1∼1.18)	1.08 (1.01∼1.17)	1.11 (0.99∼1.20)	–0.78	0.436
Phosphorous (mmol/L)	1.76 (1.41∼2.21)	1.62 (1.37∼2.06)	1.93 (1.57∼2.3)	–4.45	0.000
Iron (μmol/L)	10.60 (8.19∼13.60)	9.70 (7.50∼13.00)	11.23 (8.60∼13.81)	–2.90	0.004
PTH (pg/mL)	237.00 (112.30∼386.00)	213.00 (103.00∼361.83)	252.50 (142.70∼417.59)	–1.40	0.162
FBG (mmol/L)	6.96 (5.65∼8.92)	6.94 (5.43∼8.85)	6.96 (5.72∼8.92)	–0.18	0.856
Ferritin (ng/mL)	105.01 (35.00∼240.75)	90.00 (35.50∼234.79)	112.05 (34.61∼247.75)	–0.17	0.866
Vitamin D (ng/mL)	20.46 (12.00∼34.96)	15.04 (11.00∼31.56)	25.91 (13.77∼36.99)	–3.71	0.000
NT-proBNP (ng/L)	3437.50 (1997.50∼10384.50)	3971.50 (2275.50∼16426.00)	3074.00 (1817.25∼6316.50)	–2.88	0.004
Lymphocyte count (10^9/L)	1.18 (0.93∼1.47)	1.19 (0.88∼1.47)	1.18 (0.94∼1.48)	–0.24	0.811
Hemoglobin (g/L)	109.02 ± 16.05	106.66 ± 17.00	111.04 ± 14.93	–2.26	0.024
CRP (mg/L)	1.69 (1.60∼5.00)	1.69 (1.60∼5.08)	1.88 (1.37∼4.44)	–0.78	0.438
Kt/V urea	1.38 (1.22∼1.56)	1.4 (1.27∼1.62)	1.34 (1.19∼1.54)	–2.56	0.011

Mean ± SD is presented for variables according to the normal distribution, while median (IQR) is presented for variables with the abnormal distribution. ALT, alanine aminotransferase; AST, aspartate aminotransferase; BMI, body mass index; BUN, blood urea nitrogen; CRP, C-reactive protein; CVD, cardiovascular disease; DBP, diastolic blood pressure; DPI, dietary protein intake; FBG, fasting blood glucose; HDL-c, high-density lipoprotein cholesterol; Kt/V urea: urea clearance index; LDL-c, low-density lipoprotein cholesterol; MAMC, mid-arm muscle circumference; NT-proBNP, N-terminal pro-B-type natriuretic peptide; PTH, parathormone; SBP, systolic blood pressure; Scr, serum creatinine; SUA, serum uric acid; HD, hemodialysis; TC, total cholesterol; TG, triglycerides.

### Risk assessment of protein-energy depletion with logistic model

**FIGURE 2:**
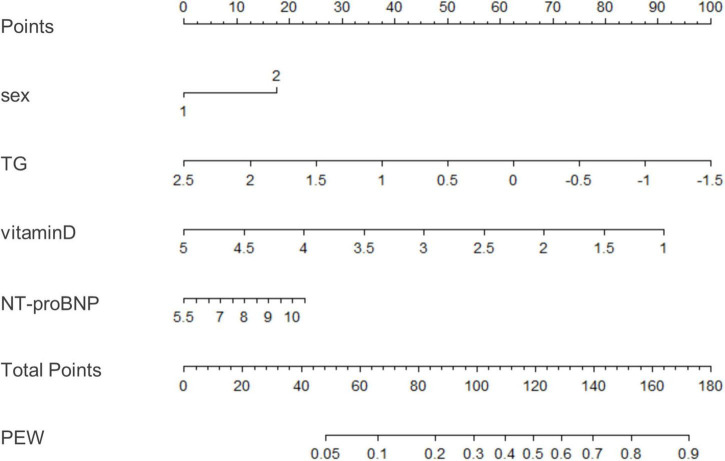
A nomogram for predicting risk of protein-energy wasting (PEW) in maintenance hemodialysis (MHD) patients. Based on the multivariate logistic regression results, the value level of each influencing factor was scored according to its contribution degree to the outcome variable. Then, each predictor was assigned a specific grading value. Finally, the predicted value of the incidence of PEW was derived from the aggregate score of four influencing factors, namely, sex, TG, vitamin D, and NT-proBNP. Sex: 1–male; 2–female. TG, triglyceride; NT-proBNP, N-terminal pro-B-type natriuretic peptide; PEW, protein-energy wasting.

We used binary logistic regression to identify significant predictors (*P* < 0.05) and then fit a model using significant predictors (*P* < 0.05) which were clinically significant. Variables of biochemical parameters were Ln-transformed to approximate normality for analysis. The data were analyzed with univariate and multivariable logistic regression to identify influencing factors of PEW (Table 4). We included all the variables with a *p*-value less than 0.05 into the multivariate logistic regression model (including monthly frequency of HD, sex, diabetes, BUN, Scr, TG, LDL-c, HDL-c, phosphorous, iron, PTH, vitamin D, NT-proBNP, hemoglobin, CRP, Kt/V urea, and ferritin). Tolerance was greater than 0.1 and variance inflation factor (VIF) was less than 10, indicating no multicollinearity (Supplementary Table 1). Based on the Akaike information criterion (AIC) results, TG (OR = 0.85, 95% CI 0.75–0.96, *P* = 0.00995) and vitamin D (OR = 0.84, 95% CI 0.72–0.98, *P* = 0.029) reduced the risk of PEW, while NT-proBNP increased the risk of PEW (*P* = 0.029, OR = 1.07, 95% CI 1.01, 1.14). Furthermore, the risk of PEW in female patients was 1.20 times higher than that in male patients (OR = 1.20, 95% CI 1.02–1.42, *P* = 0.03). According to the results of the logistic regression, we built a novel prediction model of PEW by combining four independent influencing factors with part of the ISRNM diagnostic criteria (albumin + TC + BMI + sex + TG + vitamin D + NT-proBNP). The nomogram was constructed by this novel model (Figure 2). Based on the multivariate logistic regression results, the value level of each influencing factor was scored according to its contribution degree to the outcome variable (the incidence of PEW). Then, each predictor was assigned a specific grading value. Finally, the predicted value of the incidence of PEW was derived from the aggregate score of four influencing factors.

**TABLE 4:** Odds ratios and 95% confidence intervals from multivariate logistic analysis of risk factors related to protein-energy depletion (PEW).

	PEW			
	Univariate analysis		Multivariate analysis	
Variables	OR (95%CI)	*p*	OR (95%CI)	*P*
Sex (Female)	1.73 (1.14–2.63)	0.010	1.20 (1.02, 1.42)	0.03
Age (per year)	1.01 (0.99–1.03)	0.537		
Monthly frequency of HD	1.13 (1.07–1.25)	0.013		
Education level		0.014		
Primary school	ref			
Junior school	0.65 (0.40–1.06)	0.082		
High school	0.61 (0.35–1.60)	0.080		
College	0.25 (0.09–0.71)	0.009		
Above college	0.12 (0.02–1.01)	0.051		
**Primary diseases**				
Hypertensive nephropathy	1.26 (0.66–2.41)	0.483		
Diabetic nephropathy	0.93 (0.59–1.49)	0.774		
Chronic glomerulonephritis	0.90 (0.58–1.38)	0.616		
Nephrotic syndrome	2.09 (0.60–7.28)	0.245		
IgA nephropathy	1.18 (0.29–4.77)	0.821		
Purpura nephritis	2.36 (0.21–26.23)	0.485		
Obstructive nephropathy	0.58 (0.05–6.49)	0.661		
Polycystic renal disease	1.03 (0.36–2.89)	0.961		
Etiology unknown	0.93 (0.57–1.51)	0.761		
Hypertension	0.78 (0.37–1.66)	0.526		
Diabetes	2.03 (1.33–3.11)	0.001		
Hyperlipidemia	0.32 (0.20–0.50)	0.000		
Stroke	2.04 (0.83–5.00)	0.12		
CVD	1.40 (0.80–2.45)	0.245		
SBP (per 10 mmHg)	1.02 (0.93–1.12)	0.672		
DBP (per10 mmHg)	1.05 (0.88–1.25)	0.596		
Dialysis duration (months)	1.00 (1.00–1.01)	0.608		
Bilirubin (μmol/L)^a^	1.44 (0.85–2.45)	0.174		
ALT (U/L)^a^	1.01 (0.73–1.39)	0.957		
AST (U/L)^a^	1.28 (0.92–1.79)	0.139		
BUN (μmol/L)^a^	0.30 (0.14–0.67)	0.003		
Scr (μmol/L)^a^	0.36 (0.19–0.69)	0.002		
SUA (μmol/L)^a^	0.81 (0.50–1.32)	0.403		
EGFR (ml/min)^a^	2.44 (1.34–4.45)	0.004		
TG (mmol/L)^a^	0.43 (0.30–0.61)	0.000	0.85 (0.75, 0.96)	0.009
HDL-c (mmol/L)^a^	3.46 (1.77–6.78)	0.000		
LDL-c (mmol/L)^a^	0.48 (0.29–0.79)	0.004		
Calcium (mmol/L)^a^	0.83 (0.10–6.99)	0.865		
Magnesium (mmol/L)^a^	1.24 (0.36–4.22)	0.735		
Phosphorous (mmol/L)^a^	0.29 (0.15–0.58)	0.000		
Iron (μmol/L)^a^	0.48 (0.28–0.82)	0.008		
PTH (pg/mL)^a^	0.94 (0.80–1.09)	0.404		
FBG (mmol/L)^a^	1.05 (0.60–1.83)	0.879		
Ferritin (ng/mL)^a^	0.98 (0.83–1.15)	0.760		
Vitamin D (ng/mL)^a^	0.51 (0.35–0.74)	0.000	0.84 (0.72, 0.98)	0.029
NT-proBNP (ng/L)^a^	1.39 (1.14–1.70)	0.001	1.07 (1.01, 1.14)	0.029
Lymphocyte count (10^9/L)^a^	0.77 (0.44–1.36)	0.370		
Hemoglobin (g/L)^a^	0.16 (0.04–0.60)	0.007		
CRP (mg/L)^a^	1.12 (0.93–1.34)	0.226		
Kt/V urea^a^	1.24 (0.76–2.02)	0.381		

^a^All laboratory indicators transformed using the formula LN(X) to approach a normal distribution. ALT, alanine aminotransferase; AST, aspartate aminotransferase; BUN, blood urea nitrogen; CI, confidence interval; CRP, C-reactive protein; CVD, cardiovascular disease; DBP, diastolic blood pressure; FBG, fasting blood glucose; HDL-c, high-density lipoprotein cholesterol; Kt/V urea, urea clearance index; LDL-c, low-density lipoprotein cholesterol; NT-proBNP, N-terminal pro-B-type natriuretic peptide; OR, odds ratio; PTH, parathormone; SBP, systolic blood pressure; Scr, serum creatinine; SUA, serum uric acid; HD, hemodialysis; TG, triglycerides.

### Model performance and validation

**FIGURE 3:**
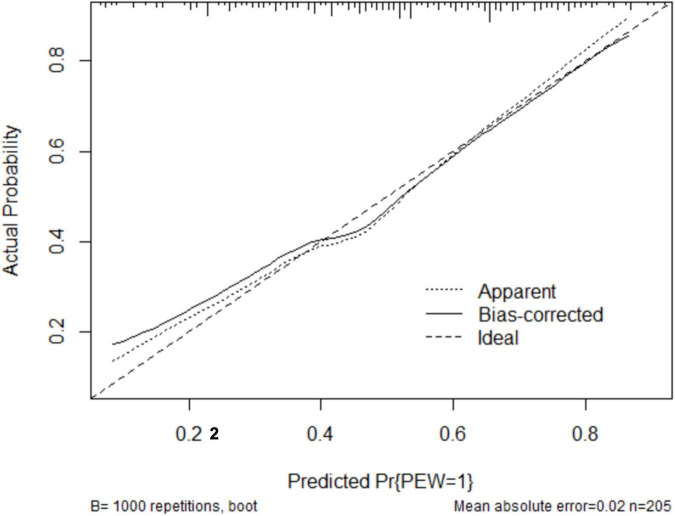
The robust performance of the nomogram in terms of consensus between the predicted risk and actual risk assessment. The clinical effectiveness of the nomogram was demonstrated by the calibration curve, which predicted the probability stratification of subjects with the bootstrap (B = 1,000) technique. The calibration curve showed good agreement between prediction and observation in the probability of PEW. PEW, protein-energy wasting.

**FIGURE 4:**
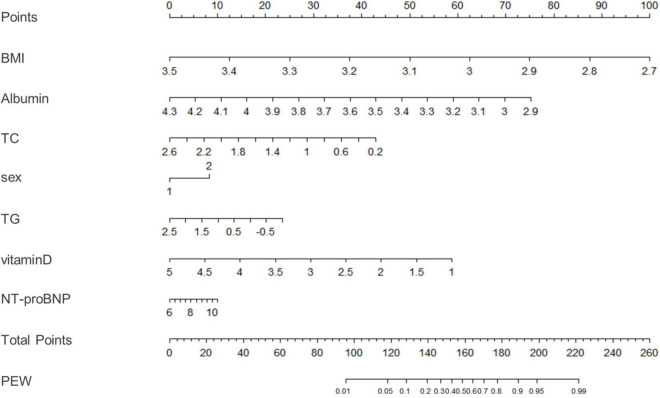
A nomogram for model 3. We used the nomogramEx package in RStudio to calculate the scores of each variable in model 3 (all variables were Ln-transformed): BMI points = –125 * BMI + 437.5; albumin points = –53.697 * albumin + 230.899; TC points = –17.863 * TC + 46.445; sex male points = 0, sex female points = 8.225; TG points = –6.719 * TG + 16.797; vitamin D points = –14.693 * vitamin D + 73.465; NT-proBNP points = 2.235 * NT-proBNP-13.411. The predicted value of the incidence of PEW was derived from the aggregate score of these influencing factors. BMI, body mass index; TC, total cholesterol; TG, triglyceride; ProBNP, pro-B-type natriuretic peptide; PEW, protein-energy wasting. *: multiply.

**FIGURE 5:**
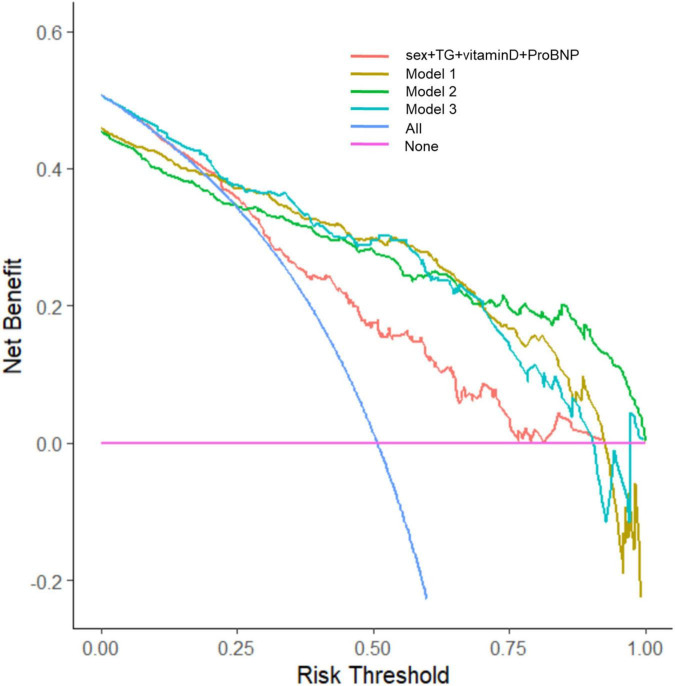
The decision curve analysis (DCA) curves of three models for diagnosing PEW. The DCA curve revealed that the net clinical benefit of model 3 was quite similar to model 1 and model 2. Model 1: albumin, TC, BMI, MAMC; model 2: albumin, TC, BMI, DPI; model 3: sex, TG, vitamin D, NT-proBNP, albumin, TC, BMI. DCA, decision curve analysis; PEW, protein-energy wasting; TC, total cholesterol; BMI, body mass index; MAMC, mid-arm muscle circumference; DPI, dietary protein intake; TG, triglyceride; NT-proBNP, N-terminal pro-B-type natriuretic peptide.

**FIGURE 6:**
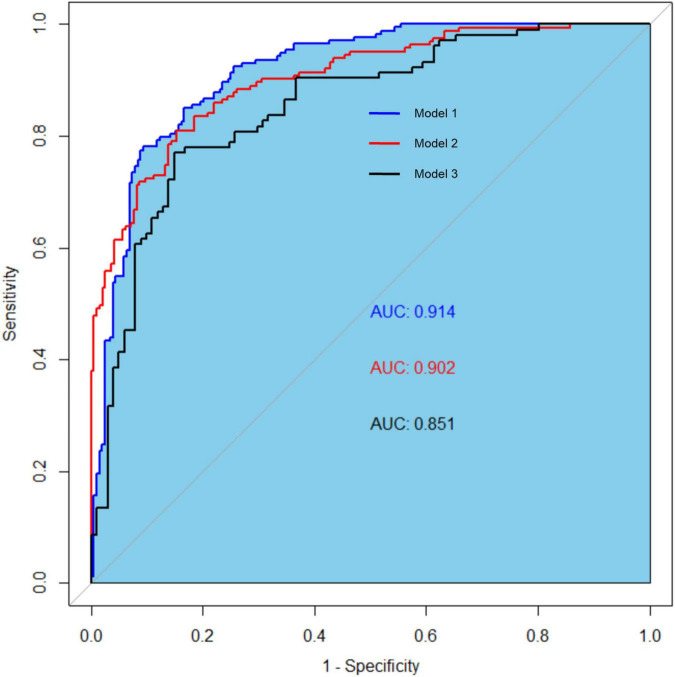
The receiver operating characteristic (ROC) curves of three models for diagnosing protein-energy wasting (PEW). The ROC curves of three models: model 1 (AUC = 0.914, 95% CI 0.886–0.943); model 2 (AUC = 0.902, 95% CI 0.871–0.933); and model 3 (AUC = 0.851, 95% CI 0.799–0.904). Model 1: albumin, TC, BMI, and MAMC; model 2: albumin, TC, BMI, and DPI; model 3: sex, TG, vitamin D, NT-proBNP, albumin, TC, and BMI. ROC, receiver operating characteristic curve; TC, total cholesterol; BMI, body mass index; MAMC, mid-arm muscle circumference; DPI, dietary protein intake; TG, triglyceride; NT-proBNP, N-terminal pro-B-type natriuretic peptide.

The clinical effectiveness of the nomogram was demonstrated by the calibration curve, which predicted the probability stratification of subjects with the bootstrap (B = 1,000) technique. The calibration curve showed good agreement between prediction and observation in the probability of PEW (Figure 3). We used the nomogramEx package in RStudio to calculate the scores of each variable in model 3 (all variables were Ln-transformed) (Figure 4): BMI points = −125 * BMI + 437.5; albumin points = −53.697 * albumin + 230.899; TC points = −17.863 * TC + 46.445; sex male points = 0, sex female points = 8.225; TG points = −6.719 * TG + 16.797; vitamin D points = −14.693 * vitamin D + 73.465; NT-proBNP points = 2.235 * NT-proBNP-13.411. The predicted value of the incidence of PEW was derived from the aggregate score of these influencing factors. We built two reference models with the ISRNM diagnosis criteria, namely, model 1 (albumin + TC + BMI + MAMC) and model 2 (albumin + TC + BMI + DPI). According to the results of the logistic regression, we built model 3 by combining four independent influencing factors with part of the ISRNM diagnostic criteria (albumin + TC + BMI + sex + TG + vitamin D + NT-proBNP). The DCA curve revealed that the net clinical benefit of model 3 was quite similar to that of model 1 and model 2 (Figure 5). In Figure 5, the abscissa was the threshold probability. When the predicted diagnostic probability reached a certain value, the PEW risk probability of patient “i” was denoted as Pi. When Pi reached a certain threshold (denoted as Pt), it was defined as positive and some intervention (such as nutritional intervention) should be taken. The horizontal line represented that all of the samples were negative without intervention, and the net benefit was “0.” The slash line indicated that all samples were positive with several interventions, and the net benefit was a backslash with a negative slope. It could be seen from the DCA curve in Figure 5 that the curve of model 3 was far away from the two extreme curves, which was within a wide Pt range, proving its high clinical practical value. Furthermore, we made a supplementary chart of clinical impact curve (Supplementary Figure 1). Model 3 was used to predict the risk stratification of 1,000 people. The “loss/benefit” coordinate axis was displayed with eight scales, and confidence intervals were displayed. The red curve (number high risk) represented the number of people classified as positive (high risk) by model 3 under each threshold probability; the blue curve (number high risk with the outcome) showed the number of true positives under each threshold probability. After comprehensive consideration of the loss–benefit ratio, it was considered that the threshold value of 60% could indicate the optimal benefit of diagnosing PEW population. Figure 6 shows the ROC curves of three models, namely, model 1 (albumin + TC + BMI + MAMC; AUC = 0.914, 95% CI 0.886–0.943); model 2 (albumin + TC + BMI + DPI; AUC = 0.902, 95% CI 0.871–0.933); and model 3 (albumin + TC + BMI + sex + TG + vitamin D + NT-proBNP; AUC = 0.851, 95% CI 0.799–0.904). A pairwise comparison of ROC curves showed that there was no difference between model 3 and model 1 or model 2 in identifying PEW (all *P* > 0.05).

## Discussion

Initiation of dialysis is an important juncture in CKD and is usually accompanied by increased mortality (28). According to the data from 11 countries, the mortality rate of CKD patients is increased in the following 120 days after first dialysis treatment (28). Although factors associated with early mortality have not been fully studied, the majority of early death can be attributed to cardiovascular events or infections, and mortality is significantly higher among PEW patients (29). PEW may increase susceptibility to catheter-related infections and cardiovascular events related to hemodynamic stress during dialysis (30). Therefore, targeted nutritional interventions need to be best implemented in the early stage of dialysis. However, the 2008 ISRNM diagnostic criteria are complex, characterized by difficulty in estimating dietary intake and assessing muscle mass loss objectively. This study aims to use clinical routine detection indicators to predict the risk of PEW in MHD patients objectively and improve the possibility of early intervention. In this research, we performed a cross-sectional study in hemodialysis patients from four different medical centers in Shanghai. Binary logistic regression was used to identify four independent influencing factors: sex, TG, vitamin D, and NT-proBNP. Then, we used these four independent variables to build a novel prediction model of PEW. The nomogram was applied to display the value of each influencing factor that contributed to PEW. ROC and DCA curves tested the prediction and discrimination ability of this novel model. Collectively, the novel prediction model could effectively identify PEW in hemodialysis patients and was more convenient than traditional diagnostic criteria.

Our analysis suggested that the incidence of PEW varied between sex in MHD patients. The female patients were presented with a significantly increased incidence of PEW compared with the male patients. This may be due to the complex distribution of adipokines in different sex, which plays an important role in energy metabolism (31–33). In addition, sex hormones may also play an important regulatory role. Compared with female patients, male patients have a higher percentage of visceral fat, and the male brain is relatively more sensitive to the catabolic effect of insulin and less sensitive to leptin than the female brain (34). Estrogen acts in the brain to increase leptin sensitivity and reduce insulin sensitivity, thus changing the distribution of body fat in female patients (34). Furthermore, several studies have demonstrated that androgens act differently in women and men. In male patients, indicators of obesity are negatively correlated with testosterone levels in all age groups (35). On the contrary, increased androgen levels will increase food intake in women, resulting in metabolic imbalance and weight gain (36–40).

Total cholesterol reflects a certain level of human energy supply (41). Our study suggested that TG played a protective role in the development of PEW. A recent study showed that plasma TG n-3 polyunsaturated fatty acids (PUFAs) are associated with lower levels of inflammatory markers and better nutritional status in MHD patients, and TG n-6 PUFAs are also associated with greater serum albumin and increased handgrip strength (20). On the contrary, TG saturated fatty acids are associated with increased insulin resistance (20), which has been proven to cause muscle wasting *via* the suppression of PI3K/Akt signaling pathway and the ubiquitin–proteasome proteolytic pathway (42). In addition, TG-monounsaturated fatty acids (MUFAs) are related to an unfavorable nutritional status, such as lower serum albumin and MAMC (20). Collectively, the role and mechanism of TG in PEW remain to be further studied.

Vitamin D plays an essential role in regulating skeletal muscle metabolism (43). It is hydroxylated to 25-hydroxyvitamin D_3_ (25-OHD_3_) in the liver and further hydroxylated to biologically active 1,25-(OH)_2_D_3_ *via* the enzyme 1-α-hydroxylase (44). CKD–MBD is prevalent in CKD patients, and high levels of PTH can induce the hydroxylation of 25-OHD_3_ to 1,25-(OH)_2_D_3_ (16). The biologically active 1,25-dihydroxyvitamin D_3_ (1,25-(OH)_2_D_3_) exerts its muscle differentiation and proliferation functions through binding with vitamin D receptor (VDR) (44). A South Korean study has shown that the average 25-(OH)D_3_ concentration in sarcopenia patients is significantly lower than that in patients without sarcopenia (45). Vitamin D deficiency is frequent among hemodialysis patients (46), and a cross-sectional study has found a positive correlation between vitamin D levels and nutritional parameters (serum albumin and serum hemoglobin) (47). Furthermore, low-level vitamin D significantly increases the mortality of MHD patients with PEW (48). These studies provide evidence that vitamin D can be a powerful PEW predictor.

Cardiac myocytes synthesize proBNP, which is mainly used as a diagnostic marker of heart failure. For MHD patients, the change of NT-proBNP may be related to non-cardiac problems, such as liquid overload, inflammation, or malnutrition (49). Recent studies have demonstrated a positive correlation between proBNP and malnutrition (50), and NT-proBNP might be an independent biomarker of PEW, especially in MHD patients (21). The accumulation of NT-proBNP is negatively associated with body fat mass and significantly correlated with the increased incidence of PEW in hemodialysis patients (51). Furthermore, NT-proBNP could predict all-cause mortality in hemodialysis patients, especially coronary heart disease (21). A recent study proposes a direction regarding how natriuretic peptides (NPs) participate in the progression of PEW (52). CKD-PEW patients are in a high catabolic state (53). Browning in high catabolic diseases such as cancer-related cachexia corresponds to the activation of brown adipocytes in white adipose tissue (53). They suggest that the uremic environment can induce browning activation, and NPs as one of the uremic toxins are involved in browning in CKD (52). This conclusion is consistent with our findings. In our research, we demonstrated that NT-proBNP had a positive correlation with the incidence of PEW.

However, there are still some limitations to our study. First, the participants included in our research are patients who have undergone hemodialysis for over 6 months; although we have collected the data from multiple hemodialysis centers, the final number of participants included is still smaller than that in the other prediction models. Second, this is cross-sectional research, and large-scale prospective studies are needed to provide more guidance information. Finally, external validation is required to confirm the reliability of the nomogram using an independent dataset. We are now expanding the database and will perform the external verification later.

In summary, we established a novel PEW prediction model by using clinical routine detection indicators (including sex, TG, vitamin D, and NT-proBNP). Compared with traditional ISRNM standard models, this novel model could avoid measurement errors in estimating dietary intake and assessing muscle mass loss and is more convenient and objective, which is helpful for clinicians to identify and intervene PEW in MHD patients in the early stage.

## Data availability statement

The datasets presented in this article are not readily available because the Management of China’s Human Genetic Resources does not allow sharing this information. Requests to access the datasets should be directed to NL, naliubrown@163.com.

## Ethics statement

The studies involving human participants were reviewed and approved by the Research Ethics Committee of Shanghai East Hospital. The patients/participants provided their written informed consent to participate in this study.

## Author contributions

NL: supervision or mentorship, research idea, and study design. SC, XM, XZh, YW, WL, LZ, XZa, YQ, YJ, SbZ, JL, HC, YS, YH, and MT: data acquisition. SC, XZh, and SgZ: statistical analysis and manuscript drafting or revision. All authors read and approved the final manuscript.

## Supplementary material

Supplementary Figure 1 The clinical impact curve of model 3 for diagnosing PEW. Model 3 was used to predict the risk stratification of 1,000 people. The “loss/benefit” coordinate axis was displayed with eight scales, and confidence intervals were displayed. The red curve (number high risk) represents the number of people classified as positive (high risk) by model 3 under each threshold probability; the blue curve (number high risk with outcome) shows the number of true positives under each threshold probability.
