# Supplementary material for: An updated clinical prediction model of protein-energy wasting for hemodialysis patients
Source: Front Nutr. 2022 Dec 6;9:933745. doi: 10.3389/fnut.2022.933745 (PMC9764006; doi:10.3389/fnut.2022.933745)
Supplement: Supplementary file 1 [file Table_1.pdf]

Table S1. Multi-collinearity analysis

variables	Collinearity Statistics	
	Tolerance	VIF
Monthly frequency of HD	0.882	1.134
Sex	0.504	1.984
Education level	0.911	1.098
Diabetes	0.798	1.254
BUN	0.586	1.706
Scr	0.579	1.726
TG	0.528	1.894
HDL-c	0.512	1.953
LDL-c	0.747	1.339
Phosphorous	0.599	1.669
Iron	0.683	1.465
PTH	0.828	1.208
Ferritin	0.737	1.356
Vitamin D	0.88	1.136
NT-proBNP	0.774	1.292
Hemoglobin	0.766	1.306
CRP	0.71	1.409
Kt/V urea	0.793	1.262

Abbreviations: BUN, blood urea nitrogen; CRP, C-reactive protein; LDL-c, low density lipoprotein cholesterol; HDL-c, high density lipoprotein cholesterol; Kt/V urea, urea clearance index; LDL-c, low density lipoprotein cholesterol; NT-proBNP, N-terminal pro-B-Type Natriuretic Peptide; PTH, parathormone; Scr, serum creatinine; TG, triglycerides; VIF, variance inflation factor.
