# Supplementary figures and images for: An updated clinical prediction model of protein-energy wasting for hemodialysis patients
Source: Front Nutr. 2022 Dec 6;9:933745. doi: 10.3389/fnut.2022.933745 (PMC9764006; doi:10.3389/fnut.2022.933745)

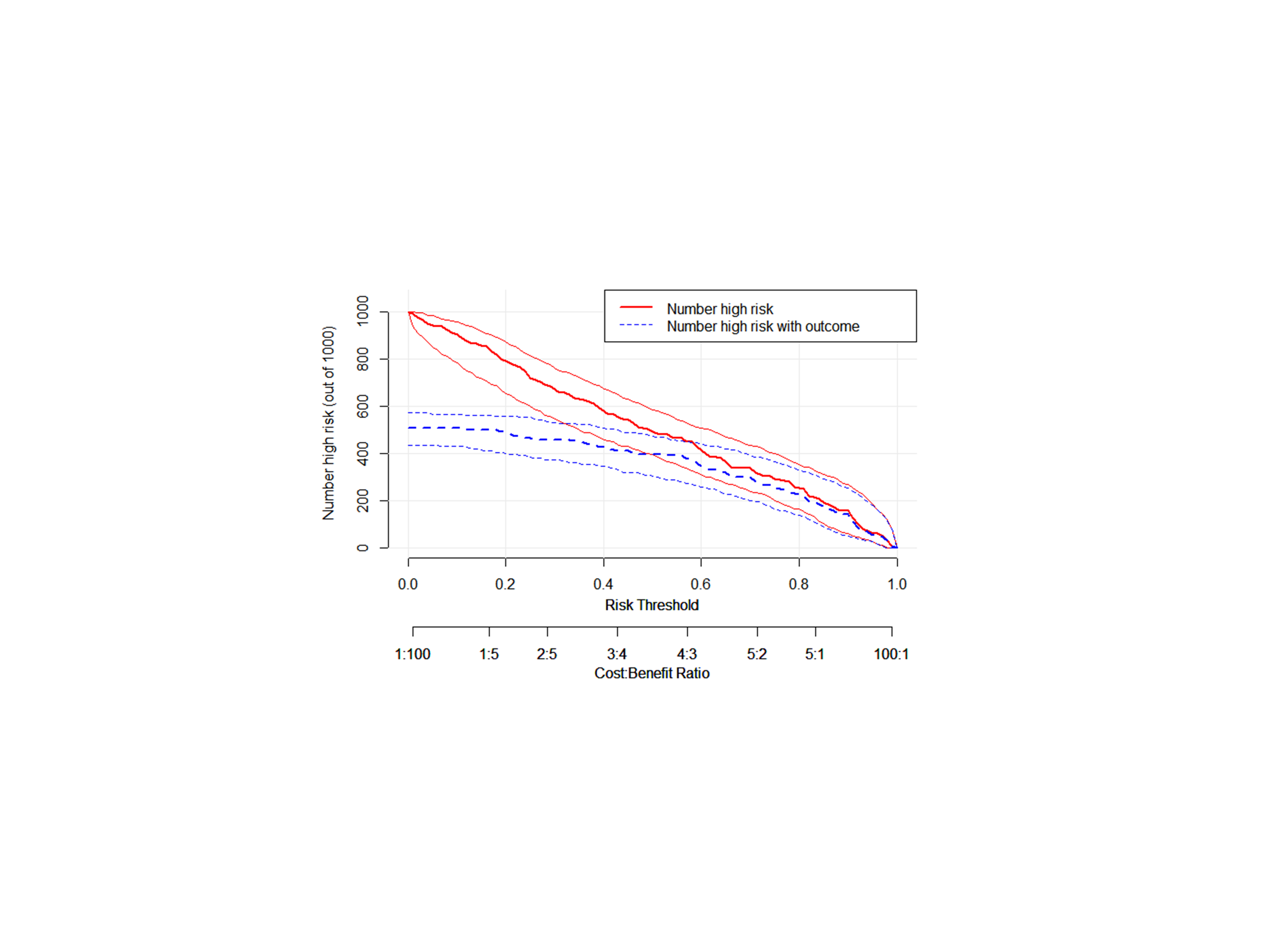

Supplement: Supplementary Figure 1 — The clinical impact curve of model 3 for diagnosing PEW. Model 3 was used to predict the risk stratification of 1,000 people. The “loss/benefit” coordinate axis was displayed with eight scales, and confidence intervals were displayed. The red curve (number high risk) represents the number of people classified as positive (high risk) by model 3 under each threshold probability; the blue curve (number high risk with outcome) shows the number of true positives under each threshold probability. [file Image_1.tif]
